# Clinical perspectives on hemoglobin measurement: The example of malaria radical cure

**DOI:** 10.1371/journal.pgph.0006614

**Published:** 2026-06-16

**Authors:** Cindy S. Chu, Eleanor Brindle, Tamiru Shibiru Degaga, Stephan Duparc, Emily Gerth-Guyette, Michelle Hsiang, Marcus Lacerda, Kamala Thriemer, Rintis Noviyanti, Dhelio Pereira, Ric Price, Leanne J. Robinson, Elisa Vidal-Cardenas, Daniel Yilma, Gonzalo J. Domingo, Stephanie Zobrist

**Affiliations:** 1 Lao-Oxford-Mahosot Hospital-Wellcome Trust Research Unit, Vientiane, Laos; 2 Centre for Tropical Medicine and Global Health, Nuffield Department of Medicine, University of Oxford, Oxford, United Kingdom; 3 Primary Health Care Program, PATH, Rockville, Maryland, United States of America; 4 College of Medicine and Health Sciences, Arba Minch University, Arba Minch, Ethiopia; 5 MMV Medicines for Malaria Venture, Geneva, Switzerland; 6 Diagnostics Program, PATH, Seatlle, Washington, United States of America; 7 Malaria Elimination Initiative, Institute for Global Health Sciences, University of California San Francisco (UCSF), San Francisco, California, United States of America; 8 Department of Epidemiology and Biostatistics, UCSF, San Francisco, California, United States of America; 9 Division of Pediatric Infectious Diseases, Department of Pediatrics, UCSF, San Francisco, California, United States of America; 10 Fundação de Medicina Tropical Doutor Heitor Vieira Dourado, Manaus, Brazil; 11 Instituto Leônidas & Maria Deane, Fiocruz, Manaus, Brazil; 12 Global and Tropical Health Division, Menzies School of Health Research, Charles Darwin University, Darwin, Australia; 13 Eijkman Research Center for Molecular Biology, BRIN, Cibinong, Indonesia; 14 Centro de Pesquisa em Medicina Tropical de Rondônia (CEPEM), Porto Velho, Brazil; 15 Mahidol Oxford Tropical Medicine Research Unit, Bangkok, Thailand; 16 Nuffield Department of Medicine, Center for Tropical Medicine and Global Health, Oxford University, Oxford, United Kingdom; 17 Burnet Institute, Melbourne, Australia; 18 Jimma University Clinical Trial Unit, Department of Internal Medicine, Institute of Health, Jimma University, Jimma, Ethiopia; Fundacao Oswaldo Cruz, BRAZIL

## Abstract

Hemoglobin (Hb) testing is widely used to inform clinical care and assess population health. A World Health Organization (WHO)-UNICEF expert group recently recommended use of venous blood tested using an automated hematology analyzer as best practice for Hb measurement in population surveys. Recently updated WHO guidelines recommend the same methods for both populations and individuals. However, in low-resource settings where these methods are often inaccessible, the performance and clinical utility of simpler methods, defined by their accuracy for key clinical decisions and impact on patient management, should be considered. We discuss Hb test performance requirements for clinical contexts as opposed to population surveys, and consider acceptability criteria specific to a test’s context and intended use. Using the example of malaria case management and radical cure, we consider the clinical risk/benefit of the incidental Hb measurement provided on a point-of-care (POC) test for glucose-6-phosphate dehydrogenase (G6PD) deficiency (the STANDARD G6PD Test), which is required to inform optimal malaria radical cure. We review evidence from clinical evaluations, where Hb was measured in venous and capillary blood using the HemoCue 201+ and the STANDARD G6PD Test, and compared to venous Hb results from an automated hematology analyzer. If Hb was not measured, radical cure could be incorrectly prescribed or withheld in more individuals with severe anemia, relative to treatment errors that could be made if any POC Hb test method were used. Overall agreement with the gold-standard Hb measure varied from 98.4% to 99.5% for identifying severe anemia. In the absence of Hb POC testing, diagnosing anemia based on clinical signs is unlikely to improve these results. We highlight the importance of evaluating the acceptable performance of POC tests by considering clinical risks/benefits specific to the intended use and health system context to improve case management where alternate solutions are unavailable or unaffordable.

## Introduction

Hemoglobin (Hb) is a biomarker widely used in anemia testing. For decades, point-of-care (POC) Hb devices, such as HemoCue® (HemoCue AB, Sweden) analyzers, have relied on capillary blood from a finger or heel prick, improving access to Hb testing for populations in whom venipuncture may be challenging, or where phlebotomists and specialized laboratories are unavailable. In low-resource settings that bear a disproportionate burden of anemia, these simple Hb measurement tools are critical.

The World Health Organization (WHO) recently recommended [1] using venous blood for measuring Hb, ideally with an automated hematology analyzer for both populations and individuals. This change arose from concern about poor reproducibility of anemia prevalence estimates from household population surveys [2–5]. Sources of imprecision in capillary POC Hb have not been identified, but training and technique likely play an important role [2,3,6]. However, recent studies and guidelines do not address diagnostic POC Hb testing by clinical staff at an individual level in low-resource settings, nor do they distinguish between clinical and survey use or define criteria for acceptable Hb data quality. Acceptable Hb variability for surveys or clinical use in low-resource contexts remains unclear.^6^ In this context, “clinically acceptable variability” is specific to a given indication for anemia testing and refers to the degree of measurement uncertainty that does not meaningfully alter the clinical decision at hand, when weighed against the risks of misclassification and available alternative testing options. The impact of these new recommendations for clinical management and outcomes in low-resource settings warrants further evaluation.

WHO concluded that precise anemia cutoffs could not be defined using the relationship between Hb and functional or clinical outcomes. Therefore, guidelines [1] relied upon statistically determined thresholds, with anemia defined by the fifth centile of Hb distributions from healthy individuals [7]. Despite the reference datasets being restricted by health status criteria, Hb concentration varied substantially, with as much as 10% difference in the fifth centile between data sources pooled to define the new thresholds [7] This indicates that there is normal variation across higher (non-anemic) Hb concentration ranges which are unlikely to be clinically meaningful. Comparisons of capillary POC to venous gold-standard Hb find that capillary imprecision can change anemia prevalence estimates, even when test bias is small, with Hb concentration differences below 10% [2–5]. These differences in the impact of Hb variability on interpretation suggest that criteria for acceptable variability should be based on the intended use (i.e., to estimate anemia prevalence or to guide clinical management) and the Hb concentration range relevant to the purpose of the test. Specifications for WHO prequalification of Hb tests [8] request information about the user and use context, but it is unclear if or how a test’s intended use might change acceptable performance criteria. Test performance metrics should align with the intended use and explicitly address the impact of any performance limitations. Any risk-benefit evaluation of using a test should also include an assessment of the counterfactual scenario where no Hb test is available, and both should be considered when establishing acceptable test performance criteria. This is particularly important given that clinical signs alone (such as tongue, conjunctival, or palmar pallor) have shown highly variable and limited overall diagnostic accuracy for detecting anemia [9,10]. Such risk-benefit assessments are necessary to support evidence-based and contextually appropriate national policy and procurement decisions regarding POC Hb testing specific to the intended use.

One example of the clinical value of POC Hb testing is malaria case management, where it is used to inform prescription of 8-aminoquinoline drugs primaquine and tafenoquine for radical cure of *Plasmodium vivax* and *Plasmodium ovale*. 8-aminoquinolines induce and exacerbate parasite-induced hemolysis and should be avoided in patients with complicated malaria and/or severe anemia [11,12]. Many national malaria treatment guidelines and clinical trials specify that patients should not be prescribed 8-aminoquinolines if they are severely anemic; exclusion thresholds range from less than 7–9 g/dL Hb. Severely anemic individuals also require specific clinical management (e.g., referral, blood transfusion, closer follow up). When malaria patients have anemia or hemolysis (either malaria or drug induced), repeated Hb measurements are necessary for clinical management.

Risks of iatrogenic hemolytic anemia following 8-aminoquinoline treatment are highest among glucose-6-phosphate dehydrogenase (G6PD)-deficient individuals. This X-linked genetic condition affects approximately 500 million people globally and can trigger acute hemolytic anemia when red blood cells encounter oxidative stress. WHO’s malaria treatment guidelines now recommend short-course primaquine or single-dose tafenoquine regimens that require quantitative POC G6PD testing [13]. Recently, POC G6PD tests have emerged that expand testing to broader populations and increase optimized and equitable access to radical cure. These tests include quantitative biosensors that present numeric G6PD enzyme activity results in U/g Hb. Because quantitative G6PD values are most reliable when normalized by either red blood cell count or Hb, POC G6PD tests ideally also measure Hb to enable normalization [14,15]. Importantly, unlike hemoglobinometers such as the HemoCue, POC G6PD tests measure both G6PD enzyme activity and Hb from the same lysate. As a result, variability in Hb measurement relative to a reference Hb assay does not necessarily equate to variability in G6PD measurement [16]. Given the utility of these diagnostics in malaria case management, the Hb result alone potentially offers clinical value, particularly in settings with no alternatives, as long as performance data substantiate its utility.

One product, the STANDARD™ G6PD Test (SD Biosensor, Republic of Korea), has recently been adopted in several malaria-endemic countries. As of 2025, the most widely available version of this test, which is approved by multiple regulatory bodies—including the Australian Therapeutic Goods Administration (TGA) and the Brazilian Health Regulatory Agency (ANVISA)—displays a Hb measurement, although it does not include a clinical indication claim for the Hb result [17]. The clinical performance of this test has been comprehensively reported by Adissu *et al.* (2023) [18]. Given its wide availability, a detailed consideration of the test’s Hb performance is warranted and should be grounded in an understanding of its clinical utility. Here, we review performance of the test as described by Adissu *et al.* [18] as a case study. We also consider the implications of these results for the test’s use in real-world malaria case management, with specific attention to identifying severe anemia (<7 g/dL for children 6–59 months and <8 g/dL for those 5 years and above [1]) as an indicator for radical cure eligibility and potentially severe malaria.

### Clinical performance of the STANDARD G6PD Test for detecting severe anemia

In Adissu *et al*. [18], capillary and venous Hb results measured with the STANDARD G6PD Test were compared against a gold-standard complete blood count on venous blood for more than 2,000 participants. Paired HemoCue 201 + data are also available. Participants included patients with suspected malaria, representative of the test’s intended use. We present a supplemental analysis of these data focused on the clinical use case of screening for severe anemia to assess radical cure eligibility. All data used in this analysis is available open source at the links listed with Adissu *et al.*

**Table 1** examines the clinical implications and risks associated with both the STANDARD G6PD and HemoCue Hb results, alongside a counterfactual scenario where a POC Hb test is not available or performed. If the capillary STANDARD G6PD Test results were used to exclude severely anemic individuals from radical cure, 92% of cases would be correctly excluded, compared to 78% by HemoCue. Misclassifications would result in 3 severely anemic individuals (8%) prescribed radical cure using the STANDARD G6PD Test, compared to 8 (22%) using the HemoCue. The lower positive predictive value (PPV) of the STANDARD G6PD Test leads to more individuals incorrectly excluded from radical cure because of its overestimation of severe anemia compared to the HemoCue. However, on capillary specimens, this occurred in only 1.5% of the total study population (34/2,231) and 0.5% using venous specimens (13/2,317) compared with 0.2% for the HemoCue for both specimen types, capillary (5/2,212) and venous (6/2167). In clinical practice, this difference translates to modest over-referral and temporary treatment delays to patients while they await confirmatory Hb testing. However, if no Hb test were performed, severe anemia would be diagnosed exclusively by symptoms and physical examination, resulting in potentially all severely anemic participants being incorrectly prescribed radical cure and increasing the risk of treatment.

**Table 1:** Theoretical quantification of risks and benefits associated with off-label clinical use of the STANDARD G6PD Test hemoglobin result to exclude individuals with severe anemia from radical cure with primaquine or tafenoquine, based on data from Adissu *et al*. [18] on capillary and venous specimens.

	*CORRECTLY WITHHELD:* Individuals who would correctly have radical cure withheld *Clinical risk:* *N/A*	*INCORRECTLY PRESCRIBED:* Individuals who would incorrectly be prescribed radical cure with primaquine or tafenoquine *Clinical risk:* *High*	*INCORRECTLY WITHHELD:* Individuals for whom radical cure with primaquine and tafenoquine would be incorrectly withheld until confirmatory testing is conducted *Clinical risk:* *Moderate (due to possible relapse)*
**CAPILLARY**			
STANDARD G6PD Test	33/36 91.7% (77.5–98.3)	3/36 8.3% (1.8–22.5)	34/2,231 1.5% (1.1–2.1)
HemoCue 201+	28/36 77.8% (60.9–89.9)	8/36 22.2% (10.1–39.2)	5/2,212 0.2% (0.1–0.5)
None: hemoglobin is not measured and/or no test is available	0 0%	36/36 (All) 100%	0 0%
**VENOUS**			
STANDARD G6PD Test	33/37 89.2% (74.6–97.0)	4/37 10.8% (3.0–25.4)	13/2,317 0.5% (0.3–1.0)
HemoCue 201+	32/37 86.5% (71.2–95.5)	5/37 13.5% (4.5–28.8)	6/2,167 0.2% (0.1–0.6)
None: hemoglobin is not measured and/or no test is available	0 0%	37/37 (All) 100%	0 0%

In **Table 2**, we present a supplemental analysis of the STANDARD G6PD Test Hb performance from Adissu *et al.* [18] using clinical value as our criteria. Capillary and venous performance of both the STANDARD G6PD Test and HemoCue for severe anemia detection is described as diagnostic sensitivity, specificity, PPV, and negative predictive value. Results demonstrate that—at minimum—the test reliabily identifies patients with severe anemia. For both capillary and venous specimens, the STANDARD G6PD Test had higher sensitivity for diagnosing severe anemia than the HemoCue. Agreement tables from Adissu *et al.* show that the STANDARD G6PD Test did not misclassify any severely anemic participants as having no or mild anemia. In comparison, the HemoCue misclassified two severely anemic participants on capillary specimens and one on venous as having no/mild anemia. However, the HemoCue had a higher overall PPV than the STANDARD G6PD Test, which tended to overestimate both moderate and severe anemia.

**Table 2:** Contingency tables and clinical performance for identification of severe anemia^&^ between the STANDARD G6PD Test and reference complete blood count (CBC) T-Hb venous measurement and the HemoCue and CBC T-Hb venous measurement for a) capillary specimens and b) venous specimens. Severe anemia by CBC is defined as a true positive. All moderate, mild and non-anemic patients by CBC are considered true negatives. Anemia categories were defined as per WHO definitions.

A Capillary specimens									
		**CBC**					**CBC**		
		Severe anemia	Moderate, mild, and no anemia	Total			Severe anemia	Moderate, mild, and no anemia	Total
**STANDARD G6PD**	Severe anemia	33	34	67	HemoCue*	Severe anemia	28	5	33
	Moderate, mild, and no anemia	3	2,197	2,200		Moderate, mild and no anemia	8	2,207	2,215
	Total	36	2,231	2,267		Total	36	2,212	2,248
Overall agreement: 98.4% (97.8–98.8)					Overall agreement: 99.4% (99.0–99.7)				
	Sensitivity	Specificity	PPV	NPV		Sensitivity	Specificity	PPV	NPV
	91.7% (77.5–98.2)	98.5% (97.9–98.9)	49.3% (36.8–61.8)	99.9% (99.6–100.0)		77.8% (60.8–89.9)	99.8% (99.5–99.9)	84.8% (68.1–94.9)	99.6% (99.3–99.8)
**B Venous specimens**									
		**CBC**					**CBC**		
		Severe anemia	Moderate, mild, and no anemia	Total			Severe anemia	Moderate, mild, and no anemia	Total
**STANDARD G6PD**	Severe anemia	33	13	46	HemoCue*	Severe anemia	32	6	38
	Moderate, mild, and no anemia	4	2,304	2,308		Moderate, mild and no anemia	5	2,161	2,166
	Total	37	2,317	2,354		Total	37	2,167	2,204
Overall agreement: 99.3% (98.8–99.6)					Overall agreement: 99.5% (99.1–99.8)				
	Sensitivity	Specificity	PPV	NPV		Sensitivity	Specificity	PPV	NPV
	89.2% (74.6–97.0)	99.4% (99.0–99.7)	71.7% (56.5–84.0)	99.8% (99.6–100.0)		86.5% (71.2–95.5)	99.7% (99.4–99.9)	84.2% (68.7–94.0)	99.8% (99.5–99.9)

& For this analysis, the WHO-established hemoglobin concentration thresholds were applied to the study population to classify participants’ anemia status, defining severe anemia as <7 g/dL for children 6–59 months and <8 g/dL for those 5 years and above [1].

* Note: Participants were only included if there were available STANDARD G6PD Test results for the relevant specimen types.

*Abbreviations:* CBC, complete blood count; G6PD, glucose-6-phosphate dehydrogenase; NPV, negative predictive value; PPV, positive predictive value; T-Hb, total hemoglobin.

It is important to note that the data discussed here were derived from controlled validation study settings with standardized procedures and trained operators, which may not fully reflect routine use in real world clinical environments. Additional data from programmatic and operational settings should be considered to further understand test performance, variability, and implementation challenges across diverse health system contexts and patient populations (e.g., children, concomitant diseases). Forthcoming results from operational research will be imperative to evaluate the test’s Hb performance under implementation conditions and provide further evidence to inform policy and procurement decisions related to malaria case management.

## Conclusions

Performance requirements for diagnostics, including POC Hb tests, should be guided by their intended clinical indications and contexts of use, and accompanied by sufficient evidence of risks and benefits to individuals. One such case is the incidental display of Hb measurement on a the STANDARD G6PD test, a tool designed to inform malaria case management. The display of Hb as auxiliary information on this test increases the capacity of providers to identify severe anemia in malaria patients, particularly in remote settings where highly qualified Hb tests are unavailable and early clinical management is essential. The STANDARD G6PD Test should not be used for exclusively measuring Hb or outside assessing eligibility for malaria radical cure. Findings from this example should not be generalized to other Hb testing applications, tests, or survey contexts. Hb test performance must be evaluated in relation to clinical risk–benefit, intended use, and patient or health system context.
